# A167 DIETARY BRANCHED-CHAIN AMINO ACIDS MEDIATE THE IMPACT OF HIGH PROTEIN DIET ON COLITIS SEVERITY THROUGH MTOR ACTIVATION

**DOI:** 10.1093/jcag/gwae059.167

**Published:** 2025-02-10

**Authors:** K Kan, L E Rondeau, P Muppidi, B Barbosa da Luz, R Dang, A Caminero

**Affiliations:** McMaster University, Hamilton, ON, Canada; McMaster University, Hamilton, ON, Canada; McMaster University, Hamilton, ON, Canada; McMaster University, Hamilton, ON, Canada; McMaster University, Hamilton, ON, Canada; McMaster University, Hamilton, ON, Canada

## Abstract

**Background:**

Dysregulated inflammation in inflammatory bowel disease (IBD) can be triggered by environmental exposures such as dietary and microbial factors. The prevalence of IBD continues to increase in Western countries, where individuals consume the western diet, characterized by high dietary intake of animal protein, fat, and sugar. Our lab has recently found that diets high in protein and animal protein promote intestinal inflammation and exacerbate colitis severity through mechanistic target of rapamycin (mTOR) activation and autophagy impairment. The mTOR pathway crucially regulates cellular growth and development through the recycling of essential components mediated by autophagy. Branched-chain amino acids (BCAAs) are potent activators of the mTOR pathway and are abundant in high protein diets and animal protein. Thus, we hypothesize that the BCAA content of these diets may contribute to the previously observed mTOR hyperactivation, autophagy inhibition, and worsened colitis severity associated with excess protein and animal protein intake.

**Aims:**

To determine the impact of dietary BCAA-enrichment on colitis severity, mTOR activation, and autophagy.

**Methods:**

Specific pathogen free C57BL/6 mice were fed a control diet (CD) (n=6/diet) or a BCAA-enriched diet (BCAA) (n=6/diet) for four weeks prior to tissue collection or three weeks prior to colitis induction. Colitis was induced through the administration of 2% w/v dextran sulfate sodium (DSS) in drinking water for 5 days, followed by 2 days of water before tissue collection. Disease activity was monitored for changes in body weight, stool consistency, and rectal bleeding, and microscopic damage scores were evaluated through histology. Colon tissues were analyzed by western blotting against mTOR proteins (p-mTOR, mTOR) and autophagy proteins (LC3-I, LC3-II). Immunohistochemistry was used to analyze colon tissue for mTOR activation levels (p-S6).

**Results:**

BCAA consumption increased levels of p-mTOR and p-S6 within colon tissue compared to the CD in groups with and without colitis. Levels of LC3-I and LC3-II decreased in groups fed the BCAA diet compared to the CD with and without colitis induction. Following colitis induction, mice fed the BCAA diet experienced increased weight loss, stool consistency, and rectal bleeding compared to mice fed the CD. BCAA consumption also elevated microscopic tissue damage compared to CD consumption.

**Conclusions:**

These findings demonstrate that BCAAs, which are enriched in high protein and animal protein diets, increase intestinal inflammation and worsen colitis severity in mice, paralleled by increased mTOR activation and impaired autophagy.

**Funding Agencies:**

CCC, CIHRCFI

